# The dynamic connectedness among infectious diseases, geopolitical risks, cryptocurrency, and commodity markets: Evidence from a partial and multiple wavelet analysis

**DOI:** 10.1371/journal.pone.0324599

**Published:** 2025-07-01

**Authors:** Hanen Ben Ameur, Fouad Jamaani, Mohammed N. Abu Alfoul

**Affiliations:** 1 Department of Economics and Finance, Taif University, College of Business Administration, Taif, Saudi Arabia; 2 Department of Computing Technologies and Data Analytics, Ezymart Corporation Pty Ltd, Sydney, Australia; Bilecik University: Bilecik Seyh Edebali Universitesi, TÜRKIYE

## Abstract

This study investigates the co-movements between prominent financial assets—crude oil, natural gas, gold, and Bitcoin—and uncertainty indices, including the Infectious Disease Equity Market Volatility Tracker (IDEMV) and the Geopolitical Risk Index (GPR), from January 2017 to January 2023. By employing advanced wavelet techniques—Wavelet Power Spectrum (WPS), Bi-Wavelet Coherence (WCA), Multiple Wavelet Coherence (MWC), and Partial Wavelet Coherence (PWC)—we analyze their time- and frequency-dependent responses to market shocks. The results reveal that Bitcoin and WTI exhibit time-varying sensitivity to IDEMV, particularly at short- and medium-term frequencies, highlighting their vulnerability to health-related crises like COVID-19. In contrast, gold and natural gas respond more strongly to GPR, with gold demonstrating a long-term leading role during geopolitical uncertainties, while Bitcoin and WTI lead in health-related shocks. The Russia-Ukraine conflict further amplified GPR’s impact on Bitcoin and increased natural gas’s vulnerability to geopolitical disruptions. These findings underscore the need for tailored strategies to address health and geopolitical risks. Policymakers should enhance crisis-response frameworks for Bitcoin and crude oil, while investors can reduce uncertainty by diversifying portfolios with resilient assets like gold and natural gas.

## Section 1: Introduction

Until the invasion of Ukraine by Russia in February 2022, the global economy was gradually recovering from the COVID-19 pandemic, which led to a huge economic shock beginning in early 2020. The ongoing Russian-Ukrainian conflict has similarly disrupted financial markets [1]. These events have increased market volatility, recession risks, and triggered energy crises, reflecting the critical role of geopolitical risk (GPR) in influencing both financial and energy markets [2].

Being the largest global producer of crude oil and the second-largest producer of natural gas [3], Russia plays a vital role in the energy supply [4]. The Ukraine conflict disrupted pipelines transporting Russian energy to Europe, exacerbating volatility in energy prices and inflation risks [5]. This geopolitical tension, compounded by the lingering effects of COVID-19, has presented severe challenges to global economic stability, further impacting markets, supply chains, and investor confidence [6].

The pandemic led to substantial declines in asset values, with Bitcoin losing 36% and WTI crude oil falling to negative prices in 2020. Energy commodities, such as WTI oil, experienced steep price drops of approximately 30% [7], reflecting heightened volatility [8]. Surprisingly, the relationship between Bitcoin and oil exhibited a significant positive correlation during the pandemic, highlighting the divergent behavior of Bitcoin compared to gold. According to [9], gold prices showed a positive trend during the pandemic, reaching a peak of $2060 and increasing by 30% in 2020. However, subsequent surges in Bitcoin’s value exposed its vulnerability to speculative bubbles [10].

The onset of the Russia-Ukraine war in 2022 marked a critical turning point, driving crude oil prices to a 14-year high of $120 per barrel [11]. Russia’s invasion of Ukraine significantly increased uncertainty across non-renewable energy markets and other major financial assets [12] underscoring the need to examine the interplay between geopolitical risks, infectious diseases, and financial markets. This study employs GPR and Infectious Disease Equity Market Volatility (IDEMV) indices to assess risks, leveraging their established relevance in measuring market impacts during crises [13,14].

Investor behavior often varies across time horizons due to differences in risk perception and expectations, contributing to heterogeneous market dynamics [15,16]. While research has explored individual effects of geopolitical risks or health crises, limited studies have examined their combined influence, particularly during successive uncertainties. This motivates our investigation into the impact of the COVID-19 pandemic and the Russia-Ukraine conflict on non-renewable energy (crude oil, natural gas), precious metals (gold), and cryptocurrency (Bitcoin).

Our study examines the lead-lag relationships between IDEMV, GPR, and major financial assets across different time scales, providing insights into how crises influence asset behavior. This study leverages advanced wavelet approaches, enabling a frequency- and time-specific analysis of co-movements, offering deeper insights than traditional models. Employing wavelet coherence methods, we evaluate co-movement patterns and capture dynamic interdependencies across investment horizons [17]. These methods allow us to identify time-frequency dependencies, revealing how financial assets respond to evolving uncertainties. In particular, we use Partial Wavelet Coherence (PWC) to examine how variables move together over time, isolating the contribution of each uncertainty factor.

This study makes three main contributions. First, by analyzing the Infectious Disease Equity Market Volatility (IDEMV) and Geopolitical Risk (GPR) indices, we address gaps in understanding how distinct sources of uncertainty impact key financial assets during the COVID-19 pandemic and the Russia-Ukraine conflict. Second, we refine existing theories on safe havens and diversification by exploring the relationships between traditional assets (gold), digital assets (Bitcoin), and commodities (WTI, natural gas) under the combined pressures of health and geopolitical shocks.

Third, through the use of partial and multiple wavelet coherence analyses, we advance dynamic connectedness research by revealing nuanced co-movement patterns and providing an updated framework for studying asset sensitivity to systematic risks. While several studies [17–19] have analyzed pairwise relationships, few have explored co-movements across multiple variables in an integrated framework. To fill this gap, our research uses partial and multiple wavelet coherence analyses to analyze co-movement dynamics through partial and multiple coherence strategies. Our findings offer practical insights for regulators and risk managers to mitigate contagion effects and improve portfolio diversification during global crises. These contributions are consistent with established financial frameworks, such as the Efficient Market Hypothesis (EMH) [20] and the Capital Asset Pricing Model (CAPM), by demonstrating how asset sensitivity to systematic risk changes across market regimes. Prior research on market efficiency and safe-haven assets [21] provide a foundation for analyzing the behavior of gold, crude oil, and bitcoin in this context.

Considering EMH and safe-haven theory, this study investigates disruptions in market efficiency and the changing roles of gold, oil, and bitcoin throughout crises. Our results provide initial evidence of different shocks combined and extensive effects on prominent financial assets during the COVID-19 pandemic and the Russia-Ukraine war. We observe that all these assets were influenced by the surge in risks, albeit on distinct scales. Wavelet Power Spectrum (WPS) results show that IDEMV fluctuates predominantly at low and medium frequencies. In 2020, an uptick in volatility was observed in the GPR Index, which was influenced by health crises and showed variations at both low and medium frequencies. These fluctuations reflect the changing levels of risk related to geopolitical risk associated with health crises over time. Moreover, with the onset of the Ukraine-Russia conflict in 2022, the GPR Index displays volatility across low, medium and high frequencies.

Using the Wavelet Coherence Approach, we highlight that the infectious disease (IDEMV) affected Bitcoin and gold returns signiﬁcantly with a negative relationship at the end of 2019, 2020−2021 (before and during COVID-19). With Bitcoin and gold, returns are leading those of IDEMV. However, post-COVID-19, the direction of the causality changes for gold, involving a positive relationship. Gold returns are lagging behind those of IDEMV. In 2022−2023 (post-COVID-19), the IDEMV index presents an important predictor of the West Texas Intermediate returns (RWTI) in the medium- and long-term scenarios. Following the COVID-19 period, GPR rose sharply in response to Russia’s invasion of Ukraine, and Bitcoin returns are leading those of GPR. In contrast, gold returns are lagging behind those of GPR.

Results indicate short-term coherence, with RWTI and GPR indices moving in phase. RGAS leads those of geopolitical risks in the long run during the Russian-Ukraine war. During the 2020–2022 post-pandemic period, fluctuations in geopolitical risk demonstrably predicted the trajectory of gas returns in the medium and long term.

By employing Multiple Wavelet Coherences, the outcomes reveal an interconnection among financial assets in the long term, highlighting a strong co-movement relationship. According to Partial Wavelet Coherences, we highlight that Bitcoin and WTI show stronger co-movement with IDEMV compared to GPR. However, natural gas and gold exhibit greater sensitivity to GPR than IDEMV over a longer period. With gold being the leader and GPR following, natural gas evolves more vulnerability to the uncertainty caused by military conflicts. Different uncertainties impact each market differently over time. The effects of COVID-19 and geopolitical risks vary in both the short and long terms. This implies that investors and risk managers should adapt their strategies based on the co-movement patterns observed across different assets.

The remainder of this paper is organized as follows: Section 2 reviews the relevant literature. Sections 3 and 4 describe the data and empirical methodology. Section 5 presents the main results and discusses their implications, and Section 6 concludes the study.

## Section 2: Literature review

Geopolitical shocks have significantly influenced global economic downturns and commodity markets over the past decade. Events such as the annexation of Crimea (2014), the Paris terror attacks (2015), U.S.-Iran tensions (2020), and the Russia-Ukraine war (2022) have disrupted global markets [18]. During the COVID-19 pandemic, lockdowns and reduced economic activities led to an oil and gas surplus, causing sharp price declines [22–24]. The energy sector faced severe damage due to oversupply and operational halts, with prices plummeting below $20 per barrel for WTI crude—the lowest in 18 years [25–27].

In addition, the ongoing conflict between Russia and Ukraine has significantly influenced global commodity markets, particularly for non-renewable energy and precious metals. Russia is a major exporter of these commodities, and any disruption to its production and export capacity can have far-reaching consequences [12].

### 2.1. General impact of uncertainty on financial markets

Recent research highlights that uncertainty—whether related to health crises, environmental issues, or financial shocks—plays a major role in shaping the behavior of financial markets. [28] show that climate-related uncertainty can significantly affect the performance of green investment indices in China, especially when linked to domestic climate policies. Similarly, [29] examine how environmental investment indices respond to major global events like the COVID-19 pandemic and the Russia-Ukraine conflict, revealing that these segments are highly sensitive to risk factors like market volatility.

Other studies point to the international transmission of uncertainty [30], find that economic policy uncertainty in the U.S. and U.K. has a stronger impact on Pakistan’s stock market volatility than domestic or Chinese uncertainty. This shows how interconnected financial markets have become, particularly during crisis periods. Likewise, [31] explore how financial stress in the U.S. affects real estate investment trusts (REITs) across Europe, Asia, and North America, using advanced frequency-domain and quantile methods. their findings emphasize the importance of monitoring stress transmission across asset classes and regions, especially during turbulent times.

### 2.2. The impact of infectious diseases on non-renewable energy, gold, and cryptocurrency markets

The impact of infectious diseases on non-renewable energy, gold, and cryptocurrency markets can exhibit variability contingent upon the disease and its implications for the world economy. The COVID-19 pandemic breakout at the start of 2020 caused significant price fluctuations within both commodity and financial markets [32].

The non-renewable energy sectors could face impacts due to infectious diseases, leading to a decline in energy demand. The pandemic’s uncertainties caused unprecedented price swings, with oil prices falling over 25% in one day [33]. Crude oil markets, vulnerable to supply-demand shocks, suffered from heightened volatility during the pandemic [8,25–27]. On the other hand, during infectious disease outbreaks that result in a severe economic downturn, investors often turn to precious metals as shelter assets. Gold is frequently perceived as a secure and reliable asset in periods of crisis, such as during the epidemics of contagious illnesses. Investors seeking to safeguard against unexpected price fluctuations in volatile markets turned to gold as a protective asset.

Gold has always served as a safe haven investment during crises, including pandemic. Investors turned to gold amid volatile markets, enhancing its role as a protective asset during COVID-19 [19,34]. More crucially, crude oil and gold are commodities often employed in portfolio allocation by fund managers and investors to diversify multiple risks [35,36]. However, evidence implies that gold’s shelter feature will likely evaporate due to market co-movements [37,38]. This ability of gold disappeared during the recent pandemic. The loss of gold’s perceived safe-haven qualities at certain times prompted people to explore alternative investment options.

Cryptocurrencies like Bitcoin are often seen as decentralized safe havens during economic instability. The rise of Bitcoin has positioned it as a notable cryptocurrency, generating substantial attention from investors and financial participants [39].

However, the uncertainty produced by COVID-19 has had a detrimental impact on Bitcoin [40,41]. Bitcoin crashed violently in November 2021, especially when the causes of past downturn markets were still valid [5]. [41,42] Argued that the volatility and unpredictability of cryptocurrencies increased during the pandemic [6] used wavelet analysis to uncover strong interconnectedness between Bitcoin returns and economic/political uncertainty during the pandemic [17]. Detected a robust negative co-movement between Bitcoin prices and the COVID-19 pandemic.

### 2.3. The impact of the geopolitical risk on the non-renewable energy, gold, and cryptocurrency markets

Geopolitical risk pertains to the potential for financial, economic, or political turmoil that may arise due to conflicts or tensions between various nations or regions. Previous research reported that geopolitical risk is detrimental to the global economy [43–46].

Geopolitical risk significantly impacts non-renewable energy markets, particularly oil and gas, due to their critical role in global affairs [45,47]. Studies confirm a strong linkage between GPR and oil price volatility [48–50]. During the Russia-Ukraine war, energy prices spiked, reflecting the vulnerability of global supply chains [51]. The global price of crude oil soared because of the supply shock, reaching its highest point in the past eight years.

Due to its unique features, the gold market was analyzed to determine the effect of GPR [52,53]. Gold can compensate for losses caused by other assets because of its negative correlation [27]. The Russia-Ukraine war demonstrated gold’s value as a refuge asset, with heightened investment during this conflict [12].

Cryptocurrencies, particularly Bitcoin, are increasingly viewed as alternatives to traditional safe-haven assets during geopolitical turmoil. Studies focus on the influence of GPR on Bitcoin’s price movements [54,55]. The Russian invasion of Ukraine introduced volatility to the Bitcoin market underscoring its sensitivity to geopolitical events [5].

### 2.4. Contributions to existing research

Our research expands the scope of existing studies by not limiting the analysisi to the COVID-19 pandemic, which has harmed the prices for a variety of commodities. Instead, we examine the effects of the ongoing Russian-Ukrainian conflict, a relatively underexplored area in the current literature. This study investigates the correlations and lead-lag relationships between the Infectious Disease Equity Market Volatility Index (IDEMV), Geopolitical Risk Index (GPR), and prominent financial assets across multiple scales and frequencies, using advanced wavelet coherence methods. Existing studies, such as, [56] concentrate on the investing tool and employ the wavelet coherence approach to determine if geopolitical risk, as a proxy for the Russia-Ukraine war, may operate as a hedge against the prices of various assets, including gold, silver, and oil. Similarly, [57] demonstrates a notable linkage between financial assets during the Russia-Ukrainian war compared to the pre-war period. It also concludes that oil plays a crucial role in transmitting the shock to other commodities and financial markets. [58] employ quantile-on-quantile regression to examine the impact of the GRP index on financial asset returns identifing both negative and positive relationships with the GPR index. Furthermore, [12] use Time-Varying Parameter Vector Autoregressive (TVP-VAR) and wavelet coherence approaches to illustrate that GPR and oil significantly transmit shocks across markets, with gas returns exhibiting higher negative spillovers.

This study builds on these insights by simultaneously exploring the interconnectedness of financial assets in the context of infectious diseases, geopolitical risks, and their combined effects on key financial assets. By examining the interplay between precious metals (gold), non-renewable energy (crude oil and gas), and cryptocurrencies (Bitcoin), our work provides a comprehensive understanding of how these uncertainties influence asset dynamics. Furthermore, the study evaluates how connectedness patterns evolve across distinct crises, offering critical implications for market participants, policymakers, and portfolio managers.

## Section 3: Econometric methodology

### 3.1. The wavelet power spectrum

The (local) wavelet spectrum of a time series x(t) is given by


$$W_{x\;}(u,s)=\;W^{x}\;(u,s)*W^{x*}\;(u,s)=\;\left\lceil W^{x}(u,s)\right\rceil \;2$$ (1)

The wavelet power spectrum gives us a measure of the local variance of the time series in the time-frequency context.

### 3.2. Wavelet coherence

Compared to traditional methods like DCC-GARCH, Granger causality, Wavelet coherence provides a key advantage: it enables simultaneous analysis in both the time and frequency domains. This allows us to detect short- and long-term co-movements, time-varying correlations, and phase differences—features especially relevant during periods of heightened uncertainty such as the COVID-19 pandemic and the Russia-Ukraine conflict.

Wavelet analysis evaluates how the spectral characteristics of a time series evolve over time, making it ideal for studying complex, crisis-driven dynamics [59]. Specifically, wavelet coherence captures relationships between Bitcoin, natural gas, gold, WTI, the GPR, and the IDEMV indices across different frequencies and time horizons. It is suitable for both stationary and non-stationary data [60] and outperforms conventional causality and correlation techniques by revealing localized co-movement patterns [61]. The method uses a bivariate framework based on the continuous wavelet transform (with the Morlet wavelet set to 6), enabling localized, multi-scale dependence analysis [62].

We estimate wavelet coherence using the cross-wavelet transform following [63], to define the co-movement of time series in both time and frequency domains.

we define the cross-wavelet transform of time-series x (t) and y (t) with the continuous wavelet transforms (CWT) $W_{n}^{x}\left(u,s\right)$ and $W_{n}^{y}\left(u,s\right)$ as follows:


$$W_{n}^{xy}(u,s)=\;W_{n}^{x}(u,s){\;W}_{n}^{y*}(u,s)$$ (2)

Where u refers to the location, whereas *s* denotes the scale, and the symbol * denotes the complex conjugate. The CWT identifies areas in the time-frequency domain where time series have a high common power; it illustrates the time series’ local covariance at each scale.

The wavelet coherence can detect co-movement between time series in the time-frequency domain. Following [64], the wavelet coherence of time-series can be defined as:


$$\begin{array}{c} R^{2}(u,s)=\;\frac{S{\;\left(s^{-1}W^{xy}(u,s)\right)|}^{2}}{S\left(S^{-1}{\left|W^{x}\;(u,s)\right|}^{2}\right)\;S\left(S^{-1}{\left|W^{y}\;(u,s)\right|}^{2}\right)} \end{array}$$ (3)

where S is considered as a smoothing operator over time as well as scale, with 0 ≤ *R*^*2*^(*u,s*) ≤ 1 [62]. The wavelet squared coherence *R*^*2*^(*u,s*) value provides a number between 0 and 1, with a high value meaning there is significant co-movement between time series and vice versa. The wavelet squared coherence only accepts positive values, unlike the standard correlation coefficient. We can find areas of co-movement between time series in the time-frequency space using the graphical representation of wavelet-squared coherence. In this circumstance, we cannot distinguish between positive and negative correlation. Consequently, we employ the Terrence and Compo phase difference [63] to offer information on positive and negative co-movements, besides causal relationships between time series. The phase difference of wavelet coherence is calculated in the following way:


$$\phi_{xy}\;(u,s)=\tan^{-1}\left(\frac{ℑ\left\{S\left(s^{-1}W^{xy}(u,s)\right)\right\}}{ℜ\left\{S\left(s^{-1}W^{xy}(u,s)\right)\right\}}\right)$$ (4)

where, ℑ and ℜ denote the Imaginary and Real portions of the smoothed cross-wavelet transform, respectively. On the wavelet coherence plots, black arrows denote the phase. A zero-phase difference indicates that the time series moves at the same time. The arrows in the visualization indicate the relative phase and correlation between the time series. A rightward-pointing arrow signifies that the time series are in-phase or positively correlated, while a leftward-pointing arrow means they are out of phase or negatively correlated. An upward-pointing arrow suggests that the first time series leads the second by π/2, whereas a downward-pointing arrow suggests that the second time series leads the first by π/2. A combination of these positions in the visualization is often observed.

### 3.3. Partial and multiple wavelet coherence

We present the partial and Multiple Wavelet Coherence (PWC and MWC) analysis developed by [65]. Using the two wavelet approaches, we examine the relationship of Bitcoin, gas, gold and WTI with the IDEMV and GRP, respectively.

The PWC and MWC methodologies are briefly described below [66]. For the given three time series, 𝑋, 𝑌, 𝑍, let 𝑊^𝑋^ (𝑆), 𝑊^𝑌^ (𝑆), 𝑊^𝑍^ (𝑆) denote their wavelet transformations. Meanwhile, 𝑊𝑌𝑋 (𝑆), 𝑊𝑌𝑍 (𝑆), and 𝑊𝑋𝑍 (𝑆) denote cross-wavelet transformations. The pairwise wavelet coherence is defined as:


$$R^{2}(X,Y)=\;\frac{{\left|S\left(s^{-1}W^{XY}(s)\right)\right|}^{2}}{S\left(s^{-1}{\left|W^{X}(s)\right|}^{2}\right)\;S\left(s^{-1}{\left|W^{Y}(s)\right|}^{2}\right)}$$ (5)

Where *S* is a smoothing operator used to balance resolution and significance.

The PWC provides wavelet coherence between two series X and Y, after removing the influence of time series Z. According to [65], the PWC is defined as follows:


$${RP}^{2}\;\left(X,Y|Z\right)=\;\frac{{\left|R(X,Y)-R(X,Z)\;R(X,Y{)}^{*}\right|}^{2}}{{\left(1-R(X,Z)\right)}^{2}\;{\left(1-R(Y,Z)\right)}^{2}}$$ (6)

Where 𝑅𝑃^2^ is the PWC squared and ranges between 0 and 1.

^*^ denotes the complex conjugation.

Similarly, the **MWC** squared is given by the following equation:


$${RM}^{2}\;(X,Y,Z)=\;\frac{R^{2}(X,Y)+\;R^{2}(X,Z)-2Re\left(R(X,Y)\;R(X,Z{)}^{*}\;R(Y,Z{)}^{*}\right)}{1-R^{2}(Y,Z)}$$ (7)

where *Re* is the smoothed cross-wavelet transform’s real part. 𝑅𝑀^2^ (𝑋, 𝑌, 𝑍) determines the coherence of various independent variables (𝑌 and 𝑍) on a dependent variable (𝑋) [67]. Monte Carlo methods were employed to estimate **PWC** and **MWC**.

## Section 4: Data

We collect daily data on Bitcoin, gold, and two non-renewable energies (crude oil and natural gas) from the Investing.com database, covering the period from January 2017 to January 2023. Prices are converted to returns by taking their logarithmic differences. Detailed descriptions of all variables, including definitions, sources, frequency, units, and direct dataset links are provided in Supporting Information (S1 Appendix).

To ensure consistency across assets, we removed non-trading days and aligned all time series by date. Minor missing values were linearly interpolated, and all data were standardized to U.S. dollars. This preprocessing step allowed for accurate comparison of return series across markets.

We selected gold and Bitcoin as the two most prominent markets. Bitcoin, the largest and most widely adopted cryptocurrency [68,69], serves as a proxy for the cryptocurrency market and represents an emerging alternative asset that attracts investor attention during periods of extreme uncertainty.

Meanwhile, gold is a widely recognized safe haven and traditional store of value, frequently analyzed in the context of geopolitical and market instability. We also include two major non-renewable energy assets: crude oil (WTI) and natural gas. Oil plays a central role in global markets and is highly sensitive to both supply-demand disruptions and geopolitical shocks. Natural gas is equally important for its energy implications, particularly in light of recent energy crises and conflicts. The combination of these four assets allows us to assess the dynamic responses of traditional (gold, oil, gas) and alternative (Bitcoin) assets to global uncertainties across both time and frequency domains.

For uncertainty indicators, we use the Daily Geopolitical Risk Index (GPR) developed by [70] which captures political and geopolitical tensions such as wars, terrorism, and state conflict. We also use the Infectious Disease Equity Market Volatility index (IDEMV) proposed by [14]. This index is published on a daily basis and is constructed through a four-category specification process. The categories include E (economic, economy, financial), M (“stock market”, equity, equities, “Standard and Poor’s”), V (volatility, volatile, uncertain, uncertainty, risk, risky), and ID (epidemic, pandemic, virus, flu, disease, coronavirus, MERS, SARS, EBOLA, H5N1, H1N1). The IDEMV index serves as an exogenous factor involving various infectious diseases. Direct dataset links for both indices are provided in Supporting Information (S1 Appendix).

We rely on daily data to capture high-frequency volatility and rapid market responses to uncertainty shocks. This frequency is particularly valuable during crises such as the COVID-19 pandemic and the Russia-Ukraine war. As emphasized by [71], daily data provide better resolution for analyzing short-term market dynamics and enhance the performance of wavelet-based time-frequency techniques.

Our dataset permits the analysis of the COVID-19 outbreak starting December 31, 2019, and supports a comparative assessment of market dynamics before and after the pandemic. It also captures the impact of the ongoing Russia-Ukraine conflict.

## Section 5: Results

### 5.1. Wavelet power spectrum

**Fig 1:**
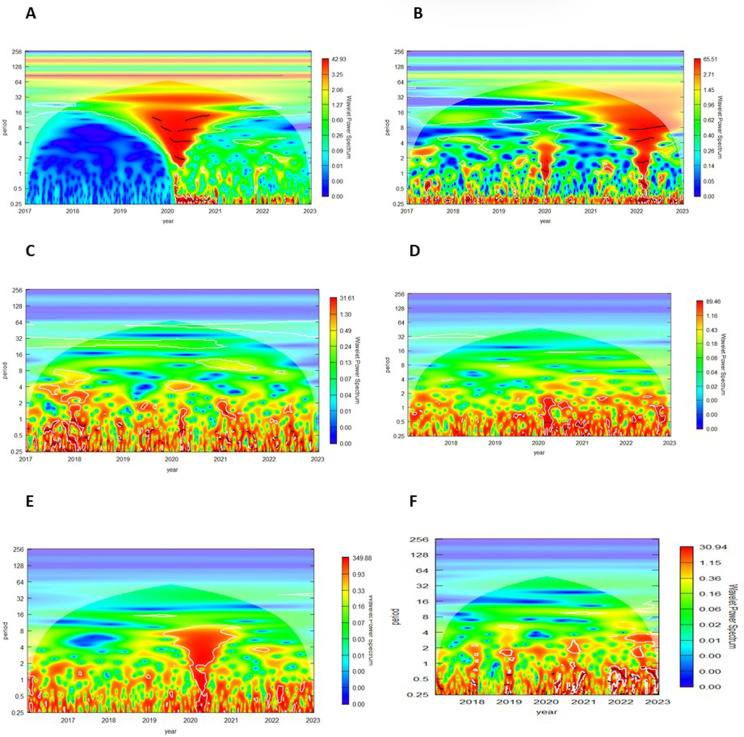
Wavelet Power Spectrum (WPS) for: (A) IDEMV, (B) GPR, (C) RBIT, (D) RGOLD, (E) RWTI, (F) RGAS.

In this section, we present Fig 1. which illustrates the Wavelet Power Spectrum (WPS) for six variables: (A) the Infectious Diseases Index (IDEMV), (B) the Geopolitical index (GPR), (C) Bitcoin returns (RBIT), (D) Gold returns (RGold), (E) West Texas Intermediate returns (RWTI), and (F) Natural GasS returns (RGAS). The WPS highlights the variance in time series across different time scales and frequencies, with significant regions at the 5% level marked by white contours.

(A) The IDEMV shows high volatility at low and medium frequencies, especially from late 2019–2021, aligning with the COVID-19 pandemic. (B) the GPR displays notable volatility at low and medium frequencies during 2020, and higher frequencies from 2022–2023, reflecting the impact of the Russia-Ukraine conflict and energy crises.

(C) Bitcoin returns exhibit high volatility at low and medium frequencies, particularly in 2018, 2020–2021, and 2022, reflecting speculative demand and market instability during crises. (D) Gold returns reveal that volatility is concentrated in low frequencies, particularly at the end of 2020–2021 and early 2023, reinforcing gold’s role as a long-term safe-haven asset.

(E) WTI crude oil returns show signiﬁcant volatility at the beginning of 2020–2021 at low and medium frequencies and at low frequencies at the beginning of 2022, in line with findings from [12].

(F) Natural Gas returns present significant volatility at low frequencies, expressly in 2019, 2020–2021, and the beginning of 2022–2023, driven by geopolitical and energy-related uncertainties. The Russian invasion of Ukraine in February 2022, as well as the accompanying European energy crisis, increased post-COVID-19 uncertainties and greatly affected the most prominent assets.

Our findings are consistent with previous studies [18,57,58] and emphasize the distinct ways financial markets react to global shocks. Bitcoin’s short-term volatility suggests its speculative behavior, while gold’s stability supports its safe-haven status. Energy markets, especially natural gas, respond strongly to geopolitical tensions, emphasizing their sensitivity to supply disruptions. By leveraging advanced wavelet techniques, this study captures time- and frequency-specific variations, offering richer insights into co-movements compared to traditional approaches.

### 5.2. Wavelet coherence approach

**Fig 2:**
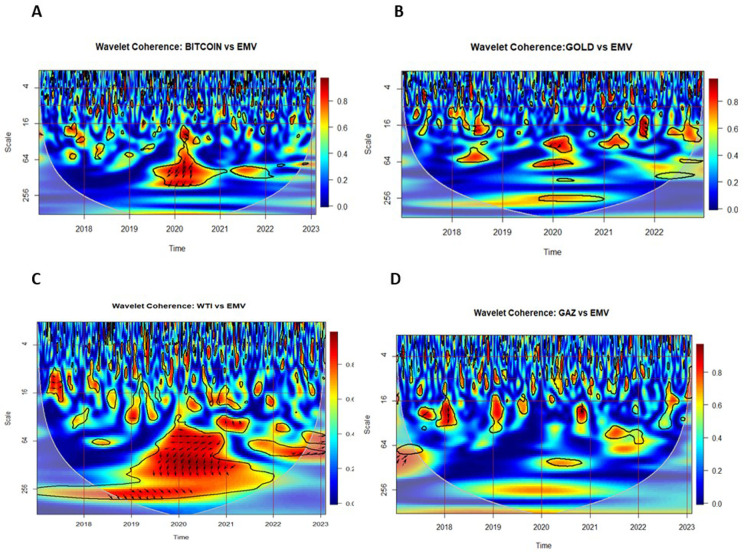
Wavelet Coherence (WC) plots for returns of Bitcoin, Gold, WTI and Natural Gas against IDEMV.

**Fig 3:**
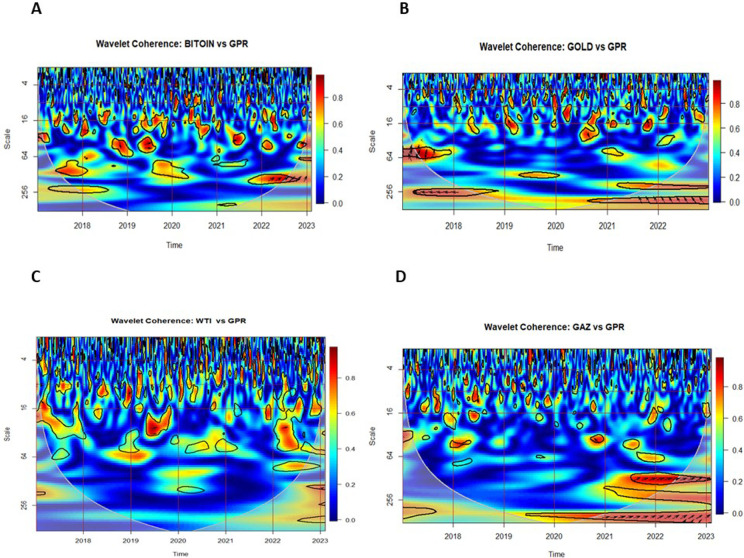
Wavelet Coherence (WC) plots for returns of Bitcoin, Gold, WTI and Natural Gas against GPR.

Figs 2 and 3 illustrate the wavelet coherence (WC) between asset returns and uncertainty indices (IDEMV and GPR). The coherence ranges from red (high) to blue (low), identifying significant co-movements at the 5% level. Arrows indicate phase relationships, revealing lead-lag dynamics.

Fig 2 displays the Wavelet Coherence (WC) between the Infectious Disease Equity Market Volatility Index (IDEMV) and the returns of four key assets: (A) Bitcoin, (B) Gold, (C) West Texas Intermediate (WTI), and (D) Natural Gas. At the top, we observe various periods of significant coherence, essentially in the 1–4 and 4–16 days throughout the crises.

(A) Bitcoin shows strong coherence at medium-and long-term frequencies (16–64 and 64–256 days). Most arrows point down and to the left (↙), indicating that IDEMV significantly affected Bitcoin returns (RBIT) with a negative relationship during late 2019 and the 2020–2021 pandemic period. This suggests that Bitcoin returns were leading those of IDEMV, consistent with the findings of [18].

(B) Gold exhibits significant short-term coherence within the 16–64 day band, especially during 2020. The arrows also point down and left-down (↙), indicating that IDEMV negatively influenced gold returns (RGOLD), with gold leading IDEMV.

However, shortly before the beginning of 2022 (post-COVID-19), the direction of the causality changes from scales 16–64, the arrows mostly point right-down, implying there is a positive relationship. These findings are supported by those of [57]. ↘ indicates gold returns are lagging those of IDEMV.

(C) WTI crude oil reveals greater co-movements with IDEMV than Natural Gas. In 2018, arrows pointing right indicate a positive medium-term relationship in the 16–64 day band. Between 2019–2021, arrows point down and left-down, showing that IDEMV negatively affected WTI returns. At longer scales (256–512 days), arrows shift to right-down. ↘ suggesting that WTI returns lagged IDEMV with a positive relationship.

In the post-COVID period (2022–2023), the coherence between IDEMV and WTI strengthens, with most arrows pointing right-up (↗), indicating a positive relationship and that IDEMV became a significant predictor of WTI returns in the medium to long term.

(D) Natural Gas displays arrows pointing down at the 16–64 day band in 2018, suggest that natural gas returns (RGAS) negatively influenced IDEMV. At the end of 2020, arrows point left-up ↖, indicating a negative relationship, with gas returns lagging IDEMV.

Fig 3 presents the Wavelet Coherence (WC) between the Geopolitical Risk Index (GPR) and the returns of four financial assets — (A) Bitcoin, (B) Gold, (C) West Texas Intermediate (WTI), and (D) Natural Gas. Significant coherence is observed at different frequencies and time periods, demonstrating how each asset responds to geopolitical shocks.

(A) Bitcoin demonstrates strong in-phase coherence during 2019–2020 in the 16–64 day frequency band. Following Russia’s invasion of Ukraine in 2022, GPR spiked sharply. During this period, Bitcoin exhibits significant coherence in the 64–256 day band, with right-up arrows (↗), indicating a positive medium- and long-term relationship, where GPR causes Bitcoin returns. ↗ also suggests that Bitcoin returns are leading those of GPR.

(B) Gold reveals that at the end of 2018, within the 256–512 day scale, arrows pointed left, denoting that the gold returns (RGOLD) and the geopolitical risk have a negative relationship in the long-term (out-of-phase), align with Shahzad et al. (2023a), who also noted gold’s long-term sensitivity to geopolitical risks. In 2019, and at the end of 2021, from scales 16–64 days, arrows pointed left-up, showing that in the medium-term, gold signiﬁcantly affected the geopolitical risks with a negative relationship. However, for the period between 2020–2021, with reference to the scales for 16–64 days, the arrows point right-down, indicating that in the medium-term, gold returns signiﬁcantly caused the geopolitical risk with a positive relationship. Finally, for scales concerning the 256–512 days, the arrows point left-up, indicating that in the long-term, the gold returns (RGOLD) signiﬁcantly affected the geopolitical risk (GPR) with a negative relationship. Gold returns are lagging behind those of GPR. These findings extend the results of [57], who observed gold’s hedging properties during geopolitical crises.

(C) WTI shows short-term coherence (16–64 days) during 2019–2020 and again in 2022. Arrows pointing right (→) indicate an in-phase, positive medium-term relationship, suggesting that WTI returns influenced GPR. This result supports earlier findings by [12] and [57] which identified oil as a transmitter of geopolitical shocks.

(D) Natural Gas indicates medium-and long-term coherence from 2020 to 2022, particularly within the 64–256 and 256–512 day frequency bands. Arrows consistently point right-up (↗), suggesting a positive relationship, with GPR acting as a strong predictor of natural gas returns during the Russia-Ukraine war. Around 2022, these arrows imply that natural gas returns led GPR,further confirming their intertwined dynamics during this conflict period.

Our findings demonstrate that Bitcoin and gold exhibited strong coherence with IDEMV and GPR during medium- and long-term periods, suggesting their sensitivity to health-related and geopolitical risks. Bitcoin’s leading role during COVID-19 implies its speculative nature, while gold’s lagging relationship reflects its traditional safe-haven characteristics. WTI’s positive relationship with IDEMV during crises underscores the vulnerability of energy markets to pandemic-induced demand shocks, whereas natural gas responded more strongly to geopolitical risks, particularly during the Russian-Ukraine war, highlighting supply-side disruptions.

### 5.3. Multiple and partial wavelet coherencies

In this section, we examine the Multiple Wavelet Coherence (MWC) and Partial Wavelet Coherence (PWC) between uncertainty indices — the Infectious Disease Equity Market Volatility Index (IDEMV) and the Geopolitical Risk Index (GPR) — and the returns of four major financial assets.

**Fig 4:**
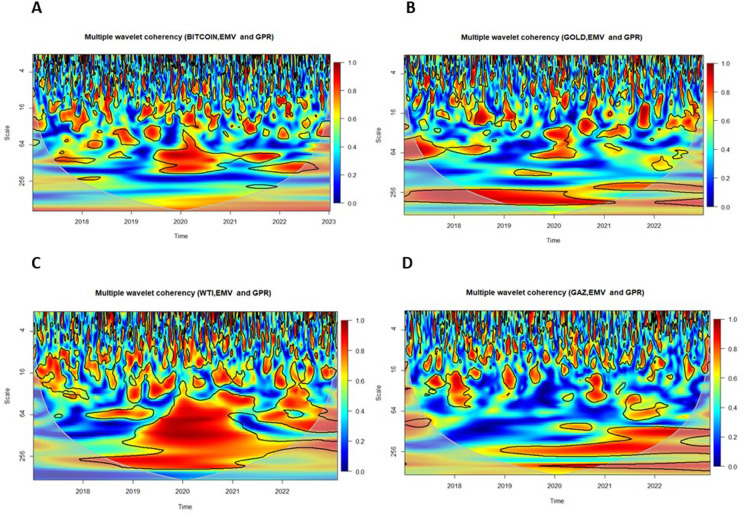
Multiple Wavelet Coherence (MWC) plots for returns of Bitcoin, gold, WTI, and natural gas with IDEMV and GPR.

Fig 4 displays the MWC between IDEMV, GPR, and the returns of key financial assets: (A) Bitcoin, (B) gold, (C) WTI crude oil, and (D) Natural Gas. These plots aim to capture the combined influence of IDEMV and GPR on cryptocurrency and commodity markets.

The horizontal axis represents the period 2017–2023, while the vertical axis represents the daily frequency. In the figures, red and blue regions indicate strong and weak co-movements, respectively. In the top and medium frequency ranges, especially towards the end of 2019 and 2020, intermittent red areas can be observed across all subfigures. In the bottom (long-term) range, red areas persist throughout the specified period for gold, WTI, and GAS. Notably, there is a strong co-movement between IDEMV, GPR, and non-renewable energies, particularly on the middle and right-hand side of the figures.

This time period they coincided with escalating trade disputes between the United States and China. Additionally, the outbreak of COVID-19 took place during this period. Therefore, the impact of GPR resulting from the trade war, Russia’s invasion of Ukraine, and the COVID-19 outbreak has significantly influenced various markets, particularly cryptocurrency, gold, and non-renewable energies.

**Fig 5:**
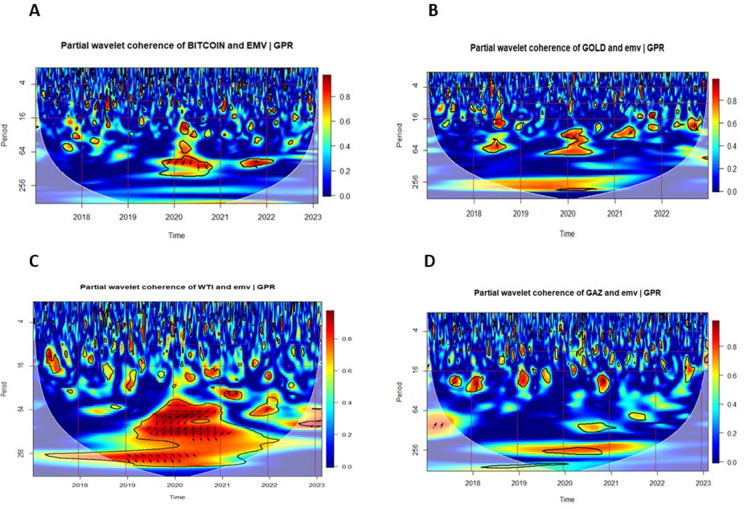
Partial Wavelet Coherence (PWC) plots with the effect of GPR removed. The color scheme in the visual representation corresponds to the wavelet coherence values, while the arrows depict the relative phase values. The coherence color code ranges from blue, indicating low or near-zero coherence, to red, representing high or close-to-one coherence. Significant coherence, determined at the 5% level, is highlighted by thick black outlines within the CoI.

**Fig 6:**
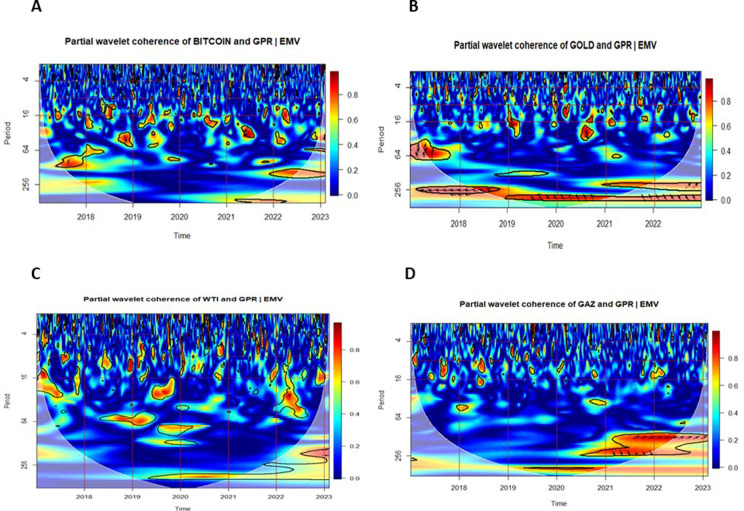
Partial Wavelet Coherence (PWC) plots with the effect of IDEMV removed. The color scheme in the visual representation corresponds to the wavelet coherence values, while the arrows depict the relative phase values. The coherence color code ranges from blue, indicating low or near-zero coherence, to red, representing high or close-to-one coherence. Significant coherence, determined at the 5% level, is highlighted by thick black outlines within the CoI.

Figs 5 and 6 illustrate the Partial Wavelet Coherence between each financial market and the uncertainty indices IDEMV and GPR. The PWC plots in Fig 5 isolate the impact of IDEMV on asset returns, removing the influence of GPR. Conversely, Fig 6 isolates the effect of GPR by removing the influence of IDEMV.

According to the findings of [72], the direction of the arrow can serve as an indicator for identifying the direction of interdependence and causality relationships. The small red regions were observed several times for the middle and high frequency (1–64 days) in all subfigures.

In Fig 5, panel A, Bitcoin returns exhibit strong medium-term coherence with IDEMV during the COVID-19 period (2020–2021), with arrows pointing left-down (↙), indicating that Bitcoin led IDEMV. In contrast, Fig 6, panel A shows weaker and more fragmented coherence with GPR, suggesting that Bitcoin was more responsive to pandemic-related uncertainty than to geopolitical shocks.

In Fig 5, panel B, Gold demonstrates short-term coherence with IDEMV in 2020–2021, again with leading behavior. In Fig 6, panel B, during 2019, in-phase coherence at long-term frequencies (256–512 days) indicates that gold moved in line with GPR. Following the onset of the Russia–Ukraine conflict, gold increasingly lagged GPR, as evidenced by medium- and long-term coherence. Arrows pointing left-up, indicating gold leads in many instances but also responds to GPR shocks post-conflict.

In Fig 5, panel C, the West Texas Intermediate returns (RWTI) reveal more co-movement with the IDEMV rather than GPR, as shown in Fig 6, panel C. This suggests that pandemic-related volatility had a greater impact on oil markets than geopolitical tensions during the sample period. Medium- and long-term co-movements appear especially significant between 2020 and 2022.

In Fig 5, panel D, Natural Gas shows limited short-term coherence with IDEMV. However, Fig 6, panel D reveals strong medium- and long-term coherence emerges with GPR, particularly from 2021 to 2023. This highlights that geopolitical risk is a major driver of natural gas volatility, especially during the Russia–Ukraine conflict. The dynamic nature of this relationship suggests that the natural gas market is more sensitive to geopolitical shocks than to health-related volatility. These results extend those of [12] and [57], offering new insights into how different markets respond asymmetrically to health-related (IDEMV) and geopolitical (GPR) uncertainties.

In fact, MWC results confirm long-term linkages between uncertainties and asset returns, while PWC isolates specific drivers of volatility. The MWC analysis highlights robust long-term interdependencies between IDEMV, GPR, and financial assets, with gold and natural gas showing the strongest co-movements during crises. The PWC analysis further reveals that Bitcoin and WTI are more sensitive to health-related uncertainties, suggesting their suitability for short-term risk hedging during pandemics. Conversely, gold and natural gas are more reactive to geopolitical risks, emphasizing their role as long-term hedges during periods of geopolitical instability.

## Section 6: Conclusions and policy recommendations

This study investigates the lead-lag relationships and co-movements between uncertainties related to infectious diseases (IDEMV), geopolitical risks (GPR), and major financial assets—including crude oil, natural gas, gold, and Bitcoin—before and after the COVID-19 pandemic and the Russia-Ukraine war. Using daily data from January 2017 to January 2023, and employing Wavelet Power Spectrum (WPS), Wavelet Coherence Analysis (WCA), Multiple Wavelet Coherence (MWC), and Partial Wavelet Coherence (PWC), we uncover time- and frequency-dependent dynamics between uncertainties and asset returns.

Our findings reveal that Bitcoin and WTI are more sensitive to IDEMV, displaying higher volatility and stronger short- and medium-term co-movements during health crises. In contrast, gold and natural gas respond more strongly to GPR, with gold acting as a long-term safe-haven asset and natural gas showing heightened vulnerability to geopolitical instability, particularly during the Russia-Ukraine war. The results further confirm that IDEMV had a significant negative impact on Bitcoin and gold returns during COVID-19, with Bitcoin leading in the short term and gold maintaining resilience over longer horizons. Post-COVID, gold began lagging IDEMV, reflecting shifts in market dynamics during the recovery phase.

Similarly, WTI and natural gas exhibited elevated volatility in response to both IDEMV and GPR, underlining their sensitivity to disruptions in energy supply and geopolitical conditions. Notably, natural gas returns led GPR during the Russia-Ukraine conflict, highlighting its strategic exposure to geopolitical shocks. MWC results confirm long-term interdependencies between uncertainty measures and asset returns, while PWC helps isolate the dominant drivers of these relationships.

Overall, our findings emphasize that financial markets exhibit time-dependent reactions to different forms of uncertainty. COVID-19 and geopolitical risks produce distinct effects across assets and time horizons. Consistent with [16], our findings affirm gold’s role as a safe haven during geopolitical turmoil, whereas Bitcoin shows limited safe-haven characteristics, particularly during health-related shocks.

These insights carry important implications. For investors, our results suggest that portfolio diversification strategies should incorporate assets like gold and natural gas, which exhibit more stable performance during crises. Conversely, assets such as Bitcoin and crude oil, while reactive, may require careful monitoring and active risk management during health shocks. For policymakers, the findings highlight the importance of adaptive risk management frameworks. For example, during pandemics, targeted stabilization efforts in the cryptocurrency and oil markets may be necessary, while geopolitical tensions may call for greater strategic focus on energy markets—especially natural gas.

Our study is subject to several limitations. While we focus on key assets, the analysis does not include equities or currency markets, which may limit the comprehensiveness of diversification insights. The relatively short sample period (2017–2023) may also restrict the ability to capture longer-term structural relationships. Additionally, macroeconomic and behavioral variables—such as inflation, interest rates, or investor sentiment—were not incorporated, though they may significantly influence asset dynamics. Finally, isolating the distinct effects of overlapping shocks (e.g., COVID-19 vs. geopolitical crises) remains analytically challenging due to their intertwined nature.

Future research could explore the asymmetric effects of uncertainty, particularly how markets respond differently to positive versus negative shocks, and how such responses vary depending on the intensity and duration of crises. This could provide deeper insight into market behavior and improve the development of targeted investment and policy strategies.

## Supporting information

S1 Appendix Data description table for all variables used in the analysis, including definitions, sources, frequency, units and direct dataset links. (DOCX)
