# Supplementary material for: The dynamic connectedness among infectious diseases, geopolitical risks, cryptocurrency, and commodity markets: Evidence from a partial and multiple wavelet analysis
Source: PLoS One. 2025 Jul 1;20(7):e0324599. doi: 10.1371/journal.pone.0324599 (PMC12212577; doi:10.1371/journal.pone.0324599)
Supplement: S1 Appendix — (DOCX) [file pone.0324599.s001.docx]

S1 Appendix:

Data Description Table

| **Variable** | **Description** | **Source** | **Period** | **Frequency** | **Currency** | **Dataset link** |
| --- | --- | --- | --- | --- | --- | --- |
| Bitcoin Price | Daily closing price of Bitcoin in USD. | Investing Database | Jan 2017 - Jan 2023 | Daily | USD | <https://www.investing.com/crypto/bitcoin/historical-data> |
| Gold Price | Daily closing price of gold per ounce in USD. | Investing Database | Jan 2017 - Jan 2023 | Daily | USD | <https://www.investing.com/commodities/gold-historical-data> |
| Crude Oil Price (WTI) | Daily closing price of West Texas Intermediate (WTI) in USD per barrel. | Investing Database | Jan 2017 - Jan 2023 | Daily | USD | <https://www.investing.com/commodities/crude-oil-historical-data> |
| Natural Gas Price | Daily closing price of natural gas in USD per MMBtu. | Investing Database | Jan 2017 - Jan 2023 | Daily | USD | <https://www.investing.com/commodities/natural-gas-historical-data> |
| Geopolitical Risk Index (GPR) | Daily index capturing geopolitical risks (wars, terrorism, tensions). | Caldara & Iacoviello (2022) | Jan 2017 - Jan 2023 | Daily | N/A | <https://www.matteoiacoviello.com/gpr.htm> |
| Infectious Disease Equity Market Volatility Index (IDEMV) | Daily index assessing the impact of pandemics on equity markets. | Baker et al. (2020) | Jan 2017 - Jan 2023 | Daily | N/A | <https://www.policyuncertainty.com/infectious_EMV.html> |
